# Biofluid Biomarkers of Alzheimer’s Disease: Progress, Problems, and Perspectives

**DOI:** 10.1007/s12264-022-00836-7

**Published:** 2022-03-19

**Authors:** Shan Huang, Yan-Jiang Wang, Junhong Guo

**Affiliations:** 1 Department of Neurology, First Hospital, Shanxi Medical University, Taiyuan, 030000 China; 2 Department of Neurology and Centre for Clinical Neuroscience, Daping Hospital, Third Military Medical University, Chongqing, 400042 China

**Keywords:** Alzheimer’s disease, Biomarker, Amyloid-beta, Tau, Diagnosis

## Abstract

Since the establishment of the biomarker-based A-T-N (Amyloid/Tau/Neurodegeneration) framework in Alzheimer’s disease (AD), the diagnosis of AD has become more precise, and cerebrospinal fluid tests and positron emission tomography examinations based on this framework have become widely accepted. However, the A-T-N framework does not encompass the whole spectrum of AD pathologies, and problems with invasiveness and high cost limit the application of the above diagnostic methods aimed at the central nervous system. Therefore, we suggest the addition of an “X” to the A-T-N framework and a focus on peripheral biomarkers in the diagnosis of AD. In this review, we retrospectively describe the recent progress in biomarkers based on the A-T-N-X framework, analyze the problems, and present our perspectives on the diagnosis of AD.

Dementia has become a global challenge with the rapid growth of the ageing population. There are 50 million people with dementia worldwide, and the number will triple by 2050 [1]. Alzheimer’s disease (AD) is the most common type of dementia and imposes substantial economic and social burdens [2]. Biomarkers are crucial for the accurate and early identification of AD and are a prerequisite for effective management of the disease. Here, we discuss the progress, problems, and perspectives of studies on biofluid biomarkers of AD.

## Addition of X to the A-T-N Biomarker Framework to Reflect the Whole Spectrum of AD Pathologies

The under-diagnosis of dementia and instability of neuropsychological evaluations are common. In addition, the patient’s medical history of cognitive and behavioral abnormalities is often obscure and uncertain. Heterogeneity in estimators, noncompliance of patients, and the floor or ceiling effect result in dissatisfaction with cognitive examinations. Sometimes, the exclusion of other neurodegenerative diseases with dementia is undefined, and the degree of matching between the clinical and postmortem diagnoses of AD is low [3]. Therefore, there are limitations regarding a clinical diagnosis of AD that is established with a medical history, cognitive examinations, and exclusions, which can diagnose only “probable” or “possible” AD and cannot reveal preclinical AD [4]. Fortunately, divergent clinical symptoms share common biomarker-associated biological mechanisms, and the categorization of patients within a biomarker-driven framework is feasible at present.

**Fig. 1:**
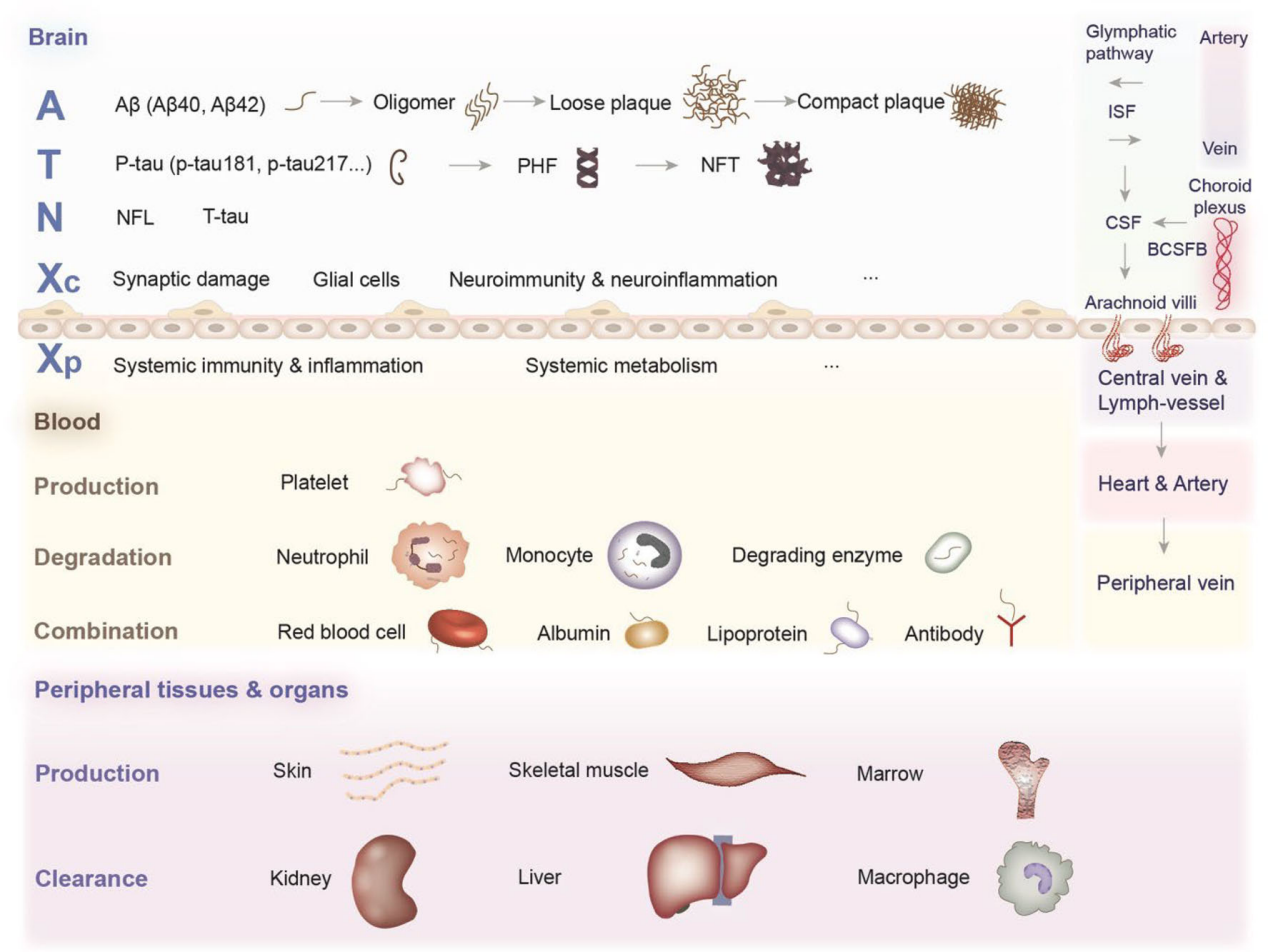
A-T-N-X framework and influencing factors in the periphery. Aβ, amyloid-beta; P-tau, phosphorylated tau; PHF, paired helical filament; NFT, neurofibrillary tangle; NFL, neurofilament light chain; T-tau, total tau; CSF, cerebrospinal fluid; ISF, interstitial fluid; BCSFB, blood-CSF barrier.

Many core biomarkers are associated with the pathology of AD. These include amyloid-beta (Aβ), pathologic tau, and markers indicating neurodegeneration, such as total tau (t-tau) and neurofilament light chain (NFL), which play important roles in the diagnosis, treatment, and prognosis of AD [5–7]. The amyloid-tangle-neurodegeneration (A-T-N) framework of biomarkers in AD was first proposed in 2016 and raised by the National Institute on Aging and Alzheimer’s Association in 2018 [8, 9]. This clinical-biological framework charts the pathophysiological features of AD and makes AD a unique disease distinguished from other neurodegenerative diseases with dementia. However, the existing framework has difficulty providing a comprehensive explanation of the pathological alterations in AD. Some pathologies and related biomarkers, such as biomarkers associated with synaptic damage, neuroinflammation, neuroimmunity, the activation of microglia and astrocytes, systemic immunity, systemic inflammation, nutrition and metabolism, apoptosis, mitochondrial dysfunction, and oxidative stress, were not included in this scheme [10–16]. “X” represents biomarkers from the abovementioned or unrealized pathologies and dynamic changes with the development of AD. Therefore, the addition of an “X” to the A-T-N framework could reflect the whole spectrum of AD pathologies and clarify the pathogenesis (Fig. 1) [17]. In our opinion, X, which is composed of heterogeneous complex systems, is neither only upstream nor downstream of A/T/N. It is worth noting that the relationship among elements in the A-T-N-X framework is an interactive and complicated network and not a simple casual cascade.

The A-T-N-X framework can also reflect different phases of AD. Simultaneously, the weights of the factors need modulation to improve the flexibility of the framework according to the phase of AD. For example, T and N play more important roles in the progression from mild cognitive impairment (MCI) to dementia [18]. In addition, the weights of factors should be adjusted at the individual level. For instance, age and sex can affect the trajectories of t-tau and NFL [19]. When there is a similar amyloid load, women are liable to have a high tau load [20–22]. Further work is needed to promote the clinical utilization of the A-T-N-X framework.

Before this framework can be widely used in clinical practice, validation, standardization, and qualification of these biomarkers are needed, and large prospective, multicenter studies are still required. We need to unify the operation of the assays and verify the appropriate criteria of the normal range to be aware of misuse and abuse. Through efforts from global consortiums on biomarkers, the methods and cut-off points of cerebrospinal fluid (CSF) biomarkers in the A-T-N-X framework have been partially aligned in AD, and the quality control program has been established [23] (https://www.alz.org/research/for_researchers/partnerships/gbsc; https://fnih.org/what-we-do/biomarkers-consortium; https://www.ifcc.org/; http://www.neurochem.info/). Evaluations of biomarkers in the framework are not limited to a simple dichotomy [24]. The use of continuous scoring systems or of more than one cut-off point can divide the biomarkers into multiple ranges, such as normal, intermediate, and abnormal ranges [9]. In addition, a standardized pre-analytical protocol has been proposed for measuring CSF biomarkers in AD [25]. These standards should be revised constantly with the development of new technologies and knowledge.

The A-T-N-X framework could also be applied in the direction of treatment and related trials. As targets of treatment, all the dimensions in the framework should be involved in cocktail therapy because the network of pathophysiology is complex and full of interconnections. As a rectification of the clinical diagnosis, the A-T-N-X framework affects inclusion and exclusion in clinical therapeutic trials. The framework could also be used to track patients. Compared with the value in diagnosis, N and X are more valuable in tracking therapeutic effectiveness and monitoring drug efficacy. In this review, we focus mainly on the application of the framework to the diagnosis of AD.

## Establishment of the Peripheral A-T-N-X Framework

When established based on pathological findings through biopsy or postmortem examination, the classical biological definition of AD is more convincing than the clinical diagnosis. According to the pathological biomarkers in the A-T-N-X framework, there are two generally accepted and well-validated approaches to the diagnosis of AD: CSF examination and positron emission tomography (PET) scans. However, CSF examination is invasive, and PET scans are costly and involve radiation exposure. Thus, the peripheral biofluid A-T-N-X system, which is more suitable for large-scale screening for AD, is urgently needed. Assays for the peripheral biofluid A-T-N-X framework have provided available alternatives, and researchers have recently focused on establishing a blood A-T-N-X system. In recent studies, the specificity and sensitivity of some blood biomarkers are comparable to those of CSF assays and PET scans in the diagnosis of AD, and these biomarkers have potential in the differential diagnosis, prognosis, and therapeutic evaluation of AD.

However, some biomarkers in the periphery, such as plasma Aβ, have shown less than satisfactory outcomes [26–28]. Here, we summarize the following challenges in the peripheral biofluid A-T-N-X framework and focus mainly on plasma biomarkers (Table 1). First, only a fraction of biomarkers from the central nervous system (CNS) enter the peripheral biofluid system through the blood-brain barrier (BBB), arachnoid granulations, glymphatic system, and the vasculature for weaker brain penetrance, and they are subsequently diluted in the bloodstream [29]. Second, within the complicated background of blood, biomarkers can be degraded by proteases or form complexes with various blood proteins or hemocytes, and these factors prohibit the accurate detection of biomarkers (Fig. 1) [30]. Third, biomarkers can be cleared in the liver and kidney and by macrophages in relevant organs, and some peripheral tissues may produce and release the same biomarkers into blood (Fig. 1) [31, 32]. Last, the levels of peripheral biomarkers fluctuate between individuals due to differences in metabolism, diet, and medication, among other factors. Furthermore, these biomarkers also fluctuate over different periods within individuals [33]. All these factors confound the association of plasma biomarkers with their counterparts in the brain.

**Table 1:** Challenges and solutions of the peripheral A-T-N-X framework.

Challenges	Solutions
Only a fraction of biomarkers from CNS enter periphery and are subsequently diluted in blood	Developing ultrasensitive technologies, advanced methods, and more accurate antibodies could improve the detection ranges of peripheral biomarkers
The transport mechanism of biomarkers from the brain to blood is still not completely clear	Evaluation of the BBB and vasculature could help to analyze the peripheral A-T-N-X system more precisely
Biomarkers can be degraded by proteases or form complexes with proteins or hemocytes in blood	NDE assays can reduce interference in blood to protect the contents from degradation
Biomarkers can be cleared or released in peripheral organs and tissues	The internal jugular vein may be an optimal blood-collection site for weakening the effect of organ clearance and blood dilution
Biomarkers in the periphery fluctuate between individuals due to differences in metabolism, diet, and medication, which also fluctuate in different periods within individuals	The methods, time of biofluid taking, and cut-off points should be unified in plasma biomarkers of AD

There are some ways to solve these challenges (Table 1). First, developing ultrasensitive technologies could improve the detection ranges of plasma biomarkers, and newly-exploited antibodies are more specific and sensitive in capturing biomarkers [34, 35]. Advanced methods for concentrating biomarkers in blood could also resolve the dilution effect. Second, neuron-derived exosomes (NDEs) are specifically derived from the CNS, and NDE assays can reduce interference in blood to protect the contents from degradation [36–38]. Third, different blood collection locations could affect the testing results. For example, the internal jugular vein may be an optimal blood collection site for weakening the effect of organ clearance and blood dilution. Fourth, BBB disruption is common in AD, and its severity differs based on disease stage and individual factors. The evaluation of BBB permeability with a unified method could help to analyze the peripheral A-T-N-X system more precisely. In addition to the BBB, the routes of biomarkers from the CNS to peripheral biofluids are still not fully understood, and require further verification. Last, on the basis of experience with mature methods in CSF biomarkers and big data analysis, we could unify the methods, time of body fluid collection, and cut-off points for plasma biomarkers of AD [6, 39, 40]. This work is developing well based on the endeavor of these global consortiums, such as the Quality Control Programme and Alzheimer’s Blood Biomarkers Program (https://www.alz.org/research/for_researchers/partnerships/gbsc). In addition, if the accuracy of the assays for biomarkers in the peripheral A-T-N-X framework cannot meet the standards of diagnosis directly, these assays can also be used as screening tools and help to make the next clinical decision.

Blood biomarkers have been widely studied, and the specificity and sensitivity of some plasma biomarkers are comparable to those of CSF assays and PET scans in the diagnosis of AD. However, the establishment of a peripheral A-T-N-X framework is not limited to blood biomarkers. Furthermore, urine, saliva, tears, and sweat are alternative non-invasive biosamples for the diagnosis of AD, and more research on biomarkers from these biosamples based on developing new technologies is needed [41, 42].

## Aβ

Aβ is a peptide containing 36–43 amino-acids that is sequentially derived from amyloid precursor protein (APP) via β-secretase and γ-secretase. Aβ is the central biomarker and the main component of amyloid plaques in AD. It is also a special protein that differs from other aberrant proteins in neurodegeneration and does not directly reflect nerve damage. Hence, Aβ is more like an upstream biomarker and apt to be applied in the early diagnosis of AD. Aβ42 is more specific in AD, and Aβ40 is known as the background of total Aβ production. The Aβ42-to-Aβ40 ratio (Aβ42/Aβ40) could balance basic Aβ production between different individuals. Aβ42 is the main component of senile plaques (SPs), and the Aβ42 oligomer is the most toxic form. SPs increase in AD, but soluble Aβ42 decreases in the CSF.

### Plasma Aβ Examination: a Milestone for the Development of AD Diagnostics

The CSF assays of Aβ40, Aβ42, their ratio, and amyloid PET have been applied in the clinic mainly for the diagnosis of AD. These approaches are well validated but restricted by the invasiveness and high costs of the procedures. Thus, researchers transferred their attention to plasma Aβ assays. In 2020, an assay for plasma Aβ, PrecivityAD™, a mass spectrometry-based assay offered by the company C2N Diagnostics, was approved for the diagnosis of AD in the USA and Europe. The consistency of PrecivityAD™ and amyloid PET is 86% (sensitivity: 92%, specificity: 76%), based on data from 686 volunteers with cognitive decline (https://www.alzforum.org/news/research-news/plasma-av-test-wins-approval-are-p-tau-tests-far-behind). Although some researchers believe the plasma assay cannot replace the examination of CSF Aβ and amyloid PET, it is still an exciting accomplishment in AD diagnostics, and the company is devoted to getting phosphorylated tau181 (p-tau181) or phosphorylated tau217 (p-tau217) into the market following this plasma Aβ assay. More efforts are needed to continually improve the accuracy of plasma Aβ measurement.

### Challenges in Plasma Aβ

Plasma Aβ has a low correlation with CSF Aβ, particularly compared to the high accuracy of phosphorylated tau (p-tau) in plasma [43]. In addition to the common challenges of the peripheral biofluid biomarkers noted above, there are some extra challenges with plasma Aβ (Table 2). First, Aβ is too sticky to flow into blood, and the transport mechanism of Aβ from brain to blood is still not completely clear. Second, in the context of blood dilution, soluble Aβ is difficult to detect in plasma, while its levels decrease further during the evolution of AD [44, 45]. Third, Aβ is derived from APP, a general membrane protein, rather than residing solely in the CNS, and has its own physiological functions, such as its role as an antimicrobial peptide. Hence, the production and clearance of Aβ in the peripheral system ^p is complex [46–48]. Fourth, owing to its amphipathic and amphoteric structure, Aβ tends to bind with various proteins and hemocytes in blood [49, 50]. Fifth, Aβ from plasma NDEs represents only intracellular Aβ in the CNS, but the main pathology of Aβ is extracellular amyloid plaques [36]. Last, while indicators such as plasma Aβ42/Aβ40 perform better, the ratio model reflects Aβ indirectly [51, 52].

**Table 2:** Challenges and solutions of plasma Aβ.

Challenges	Solutions
Aβ is too sticky to flow into blood, and the transport mechanism of Aβ from brain to blood is still not completely clear	The routes of Aβ from CSF to blood and the influencing factors within these routes should be determined
Under the background of blood dilution, soluble Aβ from the CNS is difficult to detect in plasma with a decreasing trend during the evolution of AD	Developing ultrasensitive technologies could improve the detection range for CNS-derived Aβ
Production and clearance of Aβ is more complex in the peripheral system	The most specific and sensitive isoforms or combinations of Aβ in plasma should be found that are highly correlated with the CNS
Owing to its amphipathic and amphoteric structure, Aβ tends to bind with various proteins and hemocytes in blood	Denaturation before assays could detect the released Aβ originally captured by the various blood proteins
Aβ from plasma NDEs represents only intracellular Aβ in the CNS, but the main pathology of Aβ is extracellular amyloid plaques	The relationship needs to be explained among intracellular Aβ, interstitial Aβ, Aβ in NDEs, and Aβ in SP.
While indicators such as plasma Aβ42/Aβ40, perform better, the ratio model reflects Aβ indirectly	The mechanism of Aβ and the main morbigenous type of Aβ should be verified in the brains of AD patients

### Solutions to these Problems

To address these challenges, we propose several solutions (Table 2). First, it is necessary to determine the routes of Aβ from CSF to blood and the influencing factors within these routes. We also need to explain the relationships among intracellular Aβ, interstitial Aβ, Aβ in NDEs, and Aβ in SPs. Second, preprocessing before detection could reduce the disturbance of the complex background of plasma. For instance, denaturation before enzyme-linked immunosorbent assay (ELISA) could detect the released Aβ originally captured by the various blood proteins [53]. Last, the main morbigenous type of Aβ in the brains of AD patients has to be verified and the specific and sensitive isoforms or combinations of Aβ in plasma that could highly reflect the CNS status must be found. At the same time, there are differences in the CNS and periphery between the expression of associated genes, such as *APP*, *BACE1* (beta-secretase 1), *BACE2*, *PSEN1* (presenilin 1), and *PSEN2* (presenilin 2), which could help to distinguish the source of Aβ[54, 55]. For example, APP_695_ and Aβ42 are mainly from the CNS, and APP_751_, APP_770_ and Aβ_40_ are mainly from the periphery [56]. Therefore, the combination of Aβ-associated gene expression could be a supplement to increase the accuracy of Aβ[57] estimation (Table 3).

**Table 3:** Biomarkers for X in the A-T-N-X framework.

X	Classification	Characteristics
X_C_	*Synaptic dysfunction* Ng GAP43 SNAP25 Synaptotagmin	Plasma biomarkers for synaptic dysfunction often cannot reflect damage to the brain, and the possible reason is their production in peripheral tissues The plasma NDEs of these biomarkers perform well
	*Glial cells, neuroinflammation, and immunity* GFAP S100B YKL-40 sTREM2	Glial cells play complex roles in AD, are involved in immunity and inflammation in the CNS, and are closely related to the pathogenesis of AD. The activation of astrocytes and microglia is common in AD, and the biomarkers for astrocytes and microglia are associated with AD
X_P_	*Systemic immunity and inflammation* Tumor necrosis factor, interleukin, immunoglobulin, and complement families *Systemic metabolism* Glucose, lipids (cholesterols, triglycerides), amino-acids, vitamins (homocysteine, vitamins A, B12, C, D, E, folate), trace elements, and bacterial metabolites (lipopolysaccharide, valerate, acetate, butyrate) *Others* Apoptosis, mitochondrial dysfunction, and oxidative stress	Biomarkers related to systematic immunity, inflammation, and metabolism and biomarkers related to apoptosis, mitochondrial dysfunction, or oxidative stress are nonspecific in AD; they are suited for the diagnosis of AD when combined with other core biomarkers, such as Aβ and tau, and are targets for the treatment of AD

## Tau

Aβ is viewed as the originating factor for AD, but its correlation with the later phase in AD is poor. We need to retrospectively identify downstream biomarkers that directly reflect later neurodegeneration. Tau protein is a product of the *MAPT* (microtubule associated protein tau) gene, playing a physiological role in stabilizing microtubules. As the main component of neurofibrillary tangles, pathological tau is considered to be a downstream protein of Aβ that reflects the extent of neuronal injury. Some species of tau look promising.

### PTMs of tau

Post-translational modifications (PTMs), commonly present in pathological tau, include truncation, phosphorylation, acetylation, methylation, ubiquitination, glycosylation, and nitration, among others. PTM sites in tau are associated with pathology and contribute to the diagnosis of AD. In addition to their connections to AD diagnosis, PTMs in tau are related to clinical outcomes *via* their enhancement of the propagation or reduction of the clearance of tau with individual variability [58].

Phosphorylation is the most common type of PTM in tau. Hyperphosphorylated tau is the leading component of neurofibrillary tangles. More than 70 types of p-tau have been found in neurodegeneration [59]. Recent studies have demonstrated that p-tau217, p-tau231, and p-tau181 in CSF or blood are relatively specific to AD and increase in its early stage [60–62].

Truncation is also an important type of PTM in tau. Proteolytic processing produces different fragments of tau before secretion into the extracellular fluid. These fragments vary in different types of neurodegenerative disease. For example, deletion of the first 150 and the last 50 amino-acids of tau usually promote pathology in AD, and N244 tau in CSF can distinguish AD from non-AD dementias [63–65].

The PTM map of tau has been applied to the diagnosis, discrimination, prognosis, and exploitation of antibodies for the examination or treatment of AD. Wesseling and colleagues systematically summarized the features of tau PTMs in the different phases of AD and found that isoforms of tau enriched in 0N and 4R accumulate more easily [66]. They also found that tau in AD has characteristics including a lack of a C-terminus, an increased negative charge in the proline-rich region, and a decreased positive charge in the microtubule-binding domain.

### High Accuracy of Certain P-Tau Markers in both CSF and Plasma

P-tau181 in CSF or plasma performs well in the diagnosis, differential diagnosis, and prognosis of AD [67–69]. P-tau181 also increases in the early phase of AD and is a sensitive biomarker applied in its early recognition [70]. As accurate as p-tau181, p-tau231 can also be used in the diagnosis of AD, and it increases earlier than p-tau181, with incipient AD pathology [22, 71]. Shortly after p-tau181, another p-tau, p-tau217, took center stage in the p-tau family. P-tau217 is considered to be the most robust among p-tau markers and led to a research boom last year. In the diagnosis of AD, the performance of CSF p-tau217 is better than that of p-tau181 (area under the receiver operator characteristic curve (AUC), 0.943 *vs* 0.914, *P* = 0.026) [72]. At the same time, CSF p-tau217 can distinguish AD from other neurodegenerative diseases with dementia, and the accuracy is superior to that of p-tau181 [73, 74].

The performance of p-tau217 in plasma is also promising, having a high accuracy in the diagnosis and differential diagnosis of AD by Aβ-PET or tau-PET as the outcome compared with plasma p-tau181 (AUC, 0.87 or 0.93 *vs* 0.76 or 0.83, *P <*0.001), and there is no significant difference between plasma p-tau217 and CSF p-tau217 using tau-PET as the outcome (AUC, 0.93 *vs* 0.96, *P =* 0.22) [75]. Moreover, p-tau217 rises in the asymptomatic phase and changes with the progression of AD, allowing prediction and early diagnosis of AD, while higher p-tau217 levels suggest a faster cognitive decline [76, 77]. Regarding the above advantages, p-tau217 is an appropriate biomarker with respect to the T in the peripheral A-T-N-X framework.

### Questions and Viewpoints on These Star Biomarkers

There are some questions about these p-tau biomarkers. First, how can the instability in different studies be explained? Why do these p-tau biomarkers have high diagnostic accuracy? Why does plasma p-tau have diagnostic accuracy nearly equal to that of CSF p-tau? We present our viewpoints on these questions below.

First, although p-tau217, p-tau231, and p-tau181 perform well in some cohorts, outcomes from certain studies are still unsatisfactory, especially in those comparing plasma p-tau181 with p-tau217 (plasma p-tau181, AUC = 0.67), and whether these outcomes are reliable deserve further investigation [60, 78]. It is possible that different pre-processing mechanisms, detection methods, and antibodies lead to differences in outcomes [79]. Hence, the specificity and sensitivity of p-tau217, p-tau231, or p-tau181 need to be verified in different cohorts under equal conditions.

Second, the intracellular p-tau217 level is lower than its extracellular level in the CNS, which suggests that certain isoforms of p-tau are selectively released [80]. P-tau217 also induces hyperphosphorylation of tau at multiple other sites, with aggravated tau fibrillization, and exacerbated cognitive damage [81]. P-tau217 can be related to Aβ and specifically increase in AD. Not only should the specificity and sensitivity of p-tau217, p-tau231, and p-tau181 confirmed in clinical cohorts but also the mechanisms underlying the high accuracy of these biomarkers should be explored, along with the exact amounts and overlaps among those tau proteins. In our view, it is not one specific PTM but multiple combinations of different PTMs in tau proteins that completely represent one specific tauopathy. he most specific and sensitive combination in AD and improved technologies are needed to test such a combination.

Based on on the Aβ origin theory in AD, Aβ can induce several specific PTMs of tau. With p-tau217 as an example, we propose the following hypotheses. Focusing on the specificity, we speculate that Aβ activates or inhibits a set of enzymes, including kinases (glycogen synthase kinase-3β, tau protein kinase I, and others) and phosphatases [protein phosphatase 1 (PP1), PP2A, PP2B, and others], and then the enzymes act on tau in a specific sequence [82]. Phosphorylation at one site may disrupt the PTMs of adjacent sites. The specific assembly and particular order of enzymes leads to a stable PTM pattern of tau in AD, which could be frequent in some given sites, such as N217 and N181, compared to non-AD. In the meantime, tau anchoring in different parts of the microtubule system has different patterns, while Aβ and the downstream enzymatic reaction could act on a particular part with a specific PTM pattern.

Third, the high accuracy of plasma p-tau has high clinical and translational value. Tau is dominantly generated in neural cells in the brain, so plasma tau may reflect neurodegeneration and loss of integrity of the BBB during disease progression. This may be one reason why plasma p-tau has higher diagnostic accuracy for AD than Aβ, which is ubiquitously generated. At the same time, the structural characteristics of the truncated patterns are similar in tau from plasma and CSF, which is an advantage of tau as a plasma biomarker of AD; this partly ensures consistency between some CSF p- tau proteins and their plasma levels [60]. The pathway of tau from CSF to blood and the related mechanisms need clarification. NDEs are suitable for use with tau , as tau is mainly localized intracellularly.

In summary, to ensure that they are not just passing fads, these star biomarkers need theoretical support. In the future, we need to explore the mechanisms of these biomarkers regarding the high accuracy, early alterations, and high consistency between CSF and plasma in AD.

## Biomarkers for Neurodegeneration

As a component of the axonal skeleton, NFL is a biomarker reflecting axonal degeneration [83]. NFL in CSF or plasma has high sensitivity, and changes its levels become evident before clinical symptoms of neurodegeneration, increasing in various neurodegenerative diseases, such as AD, amyotrophic lateral sclerosis, spinal muscular atrophy, multiple sclerosis, and Parkinson’s disease (PD) [84]. It is also a biomarker whose levels differ among all stages of AD and can be used to monitor its process [85]. T-tau is a biomarker of neurodegeneration that reflects the secretion of tau from neurons and nonspecific changes in cortical thickness, but it is not a direct biomarker of neuron loss [86, 87]. T-tau increases in different tauopathies, such as frontotemporal dementia (FTD), corticobasal degeneration, and progressive supranuclear palsy. In AD, t-tau is often used in ratios with other biomarkers of AD to reduce the neurodegenerative background and improve specificity in the diagnosis of AD [88, 89]. Visinin-like protein 1 (VILIP-1) is a Ca^2+^-sensor protein expressed in neurons [90]. As a biomarker of AD, VILIP-1 reflects neuronal injury, which decreases in the brain and increases in CSF in parallel with p-tau and t-tau [91].

These biomarkers can reflect the extent of severity in the late stage and the prognosis of AD. However, the above biomarkers of neurodegeneration are commonly sensitive but not specific to AD and should be used to diagnose AD in combination with specific biomarkers such as Aβ. We also explored the mechanism in the matches of the specific damaged region with different types of neurodegenerative disease.

## Biomarkers for “X”

We have divided X into two parts: X in the CNS (X_C_) and X in the periphery (X_P_). In X_C_, we focus on biomarkers associated with synaptic damage, glial cells, neuroinflammation, and immunity, and in X_P_, we focus on biomarkers associated with systemic immunity, inflammation, and metabolism.

### Biomarkers for Synaptic Dysfunction

The synapse is the basic structure of learning and memory, and synaptic loss is associated with cognitive decline. Some biomarkers of synaptic dysfunction are related to AD. The dendritic protein neurogranin (Ng) is a postsynaptic protein associated with protein kinase C. It is found mainly in neurons of the hippocampus and cortex and can bind to calmodulin and regulate long-term potentiation [92]. Ng is a promising biomarker of AD with high sensitivity and specificity and is associated with AD-specific neurodegeneration and synaptic dysfunction [93]. CSF Ng is increased in AD associated with CSF t-tau, p-tau181, and Aβ42/Aβ40, and there is no prominent change in Ng in non-AD neurodegeneration. Ng can be applied not only to the diagnosis and differential diagnosis of AD but also to the prediction of cognitive decline [94, 95]. Researchers have suggested that Ng is a specific biomarker of AD because it is a downstream protein of Aβ.

Presynaptic proteins can also be biomarkers of AD. Neuromodulin (GAP43) is indispensable for maintaining synapses and regenerating neurites, and its levels are decreased in the brain and increased in the CSF of AD. CSF GAP43 is positively correlated with Aβ deposition and tau pathology, a good performance in the diagnosis of AD (AD *vs* controls, AUC: 0.92) [96, 97]. Synaptosomal-associated protein 25 (SNAP25) is involved in vesicle fusion and exocytosis. CSF SNAP25 increases in AD, and SNAP25 1–40 can be used not only for diagnosis (AD *vs* controls, AUC: 0.93) but also for differential diagnosis (AD *vs* other dementia, AUC: 0.92) [96]. As a proxy for presynaptic Ca^2+^-sensor proteins, synaptotagmin plays an important role in exocytosis and transmitter release in the hippocampus; it also increases in CSF and could be a biomarker of AD [98].

However, plasma biomarkers for synaptic dysfunction often do not reflect damage to the brain, and a possible reason is their production in peripheral tissues [99]. Although the performance of the direct testing of synaptic biomarkers in plasma is unsatisfactory, the plasma NDEs of these biomarkers perform well, and they are worthy of further exploration for clinical application as one part of X in the peripheral A-T-N-X framework [37, 99, 100].

### Biomarkers for Glial Cells, Neuroimmunity, and Neuroinflammation

Glial cells are important for maintaining the structural integrity of neurons and homeostasis in the CNS. Astrocytes provide energy and metabolic support for neurons, and they are involved in immunity and inflammation in the CNS. Microglia are derived from the monocyte-macrophage system and are associated with neuroinflammation and immunity in the CNS. Glial cells play complex roles in AD and are closely involved in the pathogenesis of AD. The activation of astrocytes and microglia is common in AD, and biomarkers for astrocytes and microglia are associated with AD [101].

Glial fibrillary acidic protein (GFAP) is a marker of astrogliosis and is associated with amyloidosis in AD, and its expression is correlated with the density of Aβ plaques [102]. GFAP is elevated in AD and can be a biomarker for its diagnosis, differential diagnosis, and prediction [22, 103, 104]. Some studies have suggested that GFAP is associated with Aβ but not tau and that its levels change in the early stage of AD [105]. GFAP is also elevated in other neurodegenerative diseases, such as FTD, PD, and Wilson disease; therefore, it is suited to diagnose AD in combination with other AD-specific biomarkers [106–109]. S100B is a Ca^2+^-binding protein mainly in astrocytes, and is also a marker of reactive astrocytes. S100B is elevated in both CSF and plasma in AD [110, 111]. Chitinase-3-like protein 1 (YKL-40) is a glycoprotein expressed mainly in astrocytes and is associated with the innate immune system and neuroinflammation in AD [112]. YKL-40 increases in both CSF and plasma in AD [113, 114]. It is also correlated with Aβ and tau and could be a target in therapies for AD [85, 115]. Triggering receptor expressed on myeloid cells-2 (TREM2) is a receptor in the microglial membrane; it interacts directly with Aβ, which restricts the pathological enhancement of Aβ and tau[116]. Soluble TREM2 is increased in AD and correlates with t-tau and p-tau181 in the CSF of Aβ-positive individuals [117, 118]. MicroRNA-425 is a neuron-specific regulator associated with the pathophysiological microenvironment of AD, such as inflammation and amyloidosis in the CNS. It is decreased in the AD brain and can be applied as an alternative biomarker of AD [119].

### Biomarkers for Systemic Immunity, Inflammation, and Metabolism

Immunity and inflammation are essential processes at play throughout the whole AD process, and the related biomarkers could be part of the X in the A-T-N-X framework. Some nonspecific peripheral biomarkers, such as the tumor necrosis factor, interleukin, immunoglobulin, and complement families, can be used to evaluate the status of inflammation in AD [120, 121]. In addition, infectious pathogens and matched antibodies may be modifiable factors of neuroinflammation and immunity that are correlated with AD [122, 123].

Many kinds of metabolic disorder, such as diabetes and hyperlipidemia, are comorbidities of AD. Corresponding plasma metabolites, including glucose, lipids, amino-acids, vitamins, and trace elements, are associated with AD. High levels of cholesterols and triglycerides are associated with AD [124, 125]. Higher blood levels of homocysteine and lower levels of vitamins A, B12, C, D, E, and folate are correlated with MCI and AD [126, 127]. Increasing evidence suggests that bacterial metabolites are closely associated with AD [128]. Amyloid PET is positively correlated with blood lipopolysaccharide, valerate, and acetate and negatively correlated with butyrate [129].

The above biomarkers associated with immunity, inflammation, and metabolism as well as apoptosis, mitochondrial dysfunction, or oxidative stress are basically not specific to AD; however, they may be suitable for its diagnosis when combined with other core biomarkers, such as Aβ and tau, and be targets for the treatment of AD. We need to explore the specific X in this field for further application in the diagnosis and treatment of AD.

## Developing Technologies for Biomarkers in AD

The most widely-used technologies for analyzing biomarkers in AD are mass spectrometry (MS) and immunoassays. In recent years, a series of new technologies have sprung up for accurately testing biomarkers in AD. Assays for peripheral biomarkers are based on classic methods or new ultrasensitive technologies, including ELISA, single-molecule array (Simoa), immunoprecipitation/MS, liquid chromatography–MS, immunomagnetic reduction (IMR), multimer detection system, reduced graphene oxide field-effect transistor, and cryo-electron microscopy (cryo-EM) [26, 60, 130–137]. Each biomarker has a suitable assay method, and researchers have finished a set of studies comparing the different technologies [138]. Janelidze and colleagues compared eight plasma Aβ42/Aβ40 assays in two independent cohorts, and found that MS performs best [139]. Koykev and the group concluded optimal matches based on a meta-analysis (IMR for Aβ, Simoa for p-tau, and IMR or Simoa (but not ELISA) for t-tau) [40].

Antibodies are the core element of immunoassays, determining their accuracy. Assays with antibodies targeting different segments (N-terminal, C-terminal or mid-domain) or phosphorylated sites of tau differ in accuracy for AD diagnosis, differential diagnosis, and prognosis [78]. Currently, researchers from different centers often exploit their own antibodies and construct immunoassays to test biomarkers of AD. Aptamer-based assays label antibody-aptamer pairs with a lower detection limit and high specificity in CSF or serum [140].

Different technologies and different antibodies lead to inconsistent outcomes, and the data cannot be combined and analyzed together, leading to wasted biological resources. We need to identify a reliable, convenient assay with accurate antibodies and set a unified standard among the different clinical centers for biomarkers of AD.

The structures of biomarkers or combinations of interacting biomarkers are important for the exploration of AD, and clear structures can be used to find new biomarkers and exploit new antibodies for diagnosis and treatment. Structural biology and the development of related techniques such as cryo-EM could help us to understand the microscopic structure of biomarkers in AD, such as Aβ and tau[141, 142]. These techniques can be used to explore the microscopic mechanisms of interactions among the relevant biomarkers, drugs, and other related molecules in AD.

## Patterns and Trajectories of Biomarkers

Dynamic processes occur in biomarkers during AD evolution. Not a single biomarker but a group of them based on the A-T-N-X framework can describe the full spectrum of AD, and each biomarker has its own value matched with one specific phase of AD [143]. Biomarker trajectories generally assess the progression of AD and partly explain the associations among these biomarkers [144]. Tau shows site-specific phosphorylation changes during the process of AD. For example, p-tau217, p-tau181, and p-tau 231 have been shown to rise originally at the start of Aβ accumulation before the change in tau-PET, and p-tau205 and t-tau begin to increase close to the onset of clinical symptoms [71, 78, 145]. Furthermore, the fact that no change in CSF p-tau has been reported in individuals with *MAPT* mutations suggests a close association between Aβ and some site-specific p-tau proteins [146].

Familial AD cohorts and Down’s syndrome cohorts are suitable to be used to explore biomarker trajectories in AD [147, 148]. Fortea and colleagues found a changing pattern of the biomarkers in Down’s syndrome, which changed in a stable order before the clinical symptoms: the first markers were CSF Aβ42/40 and plasma NFL; the second marker was amyloid PET; the third markers were 18F-fluorodeoxyglucose PET and CSF p-tau; and the last markers were hippocampal atrophy and cognitive decline. This pattern can also be applied to sporadic and familial AD [149].

Palmqvist and colleagues described the trajectories of seven CSF and six plasma biomarkers from 337 participants in the BioFINDER study, with the accumulation of amyloid deposition in AD. They found two main outcomes. The first was that the matching biomarkers in CSF and plasma began to change almost simultaneously, but the dynamic ranges in plasma were smaller than those in CSF, except for p-tau (similar in plasma and CSF). The second was the sequential order, in the sequence of Aβ, soluble p-tau, and biomarkers related to “N” and inflammation. This pattern supported the theory of the amyloid cascade in AD and suggested that inflammation should be included as the “X” (in the A-T-N-X framework) in the central and peripheral systems [45].

Significantly, certain cross-sections of A-T-N-X could be used to determine the prognosis of AD. For example, there is a more rapid decline in patients with A(+)-T(+)-N(+) than other profiles [150, 151]. Extrapolating from these outcomes, T(+) or N(+) indicates a steeper exacerbation of AD based on A(+) [152].

Following the trajectories of these studies, we found an objective relationship among these biomarkers. In addition, we raise several hypotheses regarding the mechanisms among biomarkers in AD. We still consider that Aβ is the central and original biomarker of AD and differentiates AD from other neurodegenerative diseases. Exploration of the interactions of the suggested biomarkers with Aβ is essential to reveal the specific meaning of each. We could ascertain the physical characteristics of combinations of Aβ and other biomarkers or antibodies with new techniques, and there could be some key domains involved in specific binding. We can also analyze the biochemical characteristics of some special molecules, enzymes, or pathways of the biomarkers. Moreover, we cannot exclude that some biomarkers are specific to AD but independent of Aβ, so we should explore the mechanisms by which these biomarkers can identify AD alone.

## Complete Model Based on the Biomarkers of AD

We seek to build a comprehensive, reliable, and available model based on biomarkers of the A-T-N-X framework, which could be applied in the diagnosis, differential diagnosis, prevention, prognosis, and treatment of AD. First, basic information, such as age, sex, body mass index, underlying disease, metabolism, nutrition, diet, exercise, education, and medications (such as cholinesterase inhibitors and memantines), should be included. Then, comorbidities and related biomarkers should be included in the whole model, as this contributes to the early diagnosis of AD in high-risk populations [153]. Second, the clinical status, especially cognitive assessment, such as the Mini-mental State Examination, Alzheimer’s Disease Assessment Scale-Cognitive Subscale, Clinical Dementia Rating, Preclinical Alzheimer’s Cognitive Composite, and Montreal Cognitive Assessment scores, should be taken into account. Third, the results of basic examinations and laboratory tests, such as magnetic resonance imaging and liver and renal function tests, should be included. Fourth, tests of the genes associated with AD, including *APP*, *BACE1*, *PSEN1*, *APOE* (apolipoprotein E) ε4 alleles, and *TREM2*, and calculation of the polygenic risk score should be performed [154]. Proteomic profiling is an alternative biomarker panel for AD, the results of which can offer directions for further exploration of new biomarkers [82, 155, 156]. Last, biomarkers should be central to the model, including the central framework (PET, CSF assays, or biopsy) and the peripheral framework (plasma or other biofluid assays). The consistency of a biomarker in CSF and plasma should be weighed individually before deciding to choose peripheral biomarkers. The whole model is helpful in early prediction and early diagnosis.

## Perspectives

With the generation-after-generation appearance of star biomarkers, we need to see through the initial excitement generated by their discovery to perceive their real importance in AD. We hope to identify true representative biomarkers that are stable and accurate, with clear mechanisms in AD. The A-T-N-X framework of AD provides a common language for investigators. In the future, we should pay more attention to the peripheral biofluid A-T-N-X framework, focusing especially on improving the accuracy of measurement of peripheral biofluid Aβ based on ultrasensitive technologies. Specifically, we should clarify the mechanisms by which the biomarkers support the peripheral A-T-N-X framework in AD. In addition, we need to unify the assay methods and cut-off points of the plasma biomarkers in multicenter studies. Clinical trials targeting biomarkers should then be further improved. Finally, we should construct a comprehensive model based on biomarkers to assess individuals suitable for further studies and applications in the clinic. This biomarker-based framework could be applied to more neurodegenerative diseases.
